# Association of Autofluorescent Advanced Glycation End Products (AGEs) with Frailty Components in Chronic Kidney Disease (CKD): Data from a Single-Center Cohort Study

**DOI:** 10.3390/cells12030438

**Published:** 2023-01-29

**Authors:** Paolo Molinari, Lara Caldiroli, Elena Dozio, Roberta Rigolini, Paola Giubbilini, Francesca Maria Ida Carminati, Giuseppe Castellano, Massimiliano M. Corsi Romanelli, Simone Vettoretti

**Affiliations:** 1 Unit of Nephrology, Dialysis and Kidney Transplantation, Fondazione IRCCS Ca’ Granda Ospedale Maggiore Policlinico di Milano, 20122 Milan, Italy; 2 Laboratory of Clinical Pathology, Department of Biomedical Science for Health, Università degli Studi di Milano, 20133 Milan, Italy; 3 Service of Laboratory Medicine1-Clinical Pathology, IRCCS Policlinico San Donato, San Donato Milanese, 20097 Milan, Italy; 4 Department of Clinical Sciences and Community Health, Università degli Studi di Milano, 20122 Milan, Italy

**Keywords:** advanced glycation end products (AGEs), chronic kidney disease (CKD), frailty, soluble receptor for AGE (sRAGE), cleaved RAGE (cRAGE), endogenous secretory RAGE (esRAGE)

## Abstract

Background: Chronic kidney disease (CKD) is characterized by an overproduction and accumulation of advanced glycation end products (AGEs). Because AGEs may play a role in the development of malnutrition and sarcopenia, two essential components of frailty, we evaluated whether they may also contribute to the onset of frailty in CKD patients. Methods: We performed a cross-sectional analysis of 117 patients. AGEs were quantified using a fluorescence spectrophotometer and soluble receptor for AGE (sRAGE) isoforms by ELISA. We defined frailty according to the frailty phenotype (FP) proposed by Fried. Results: The average age of patients was 80 ± 11 years, 70% were male, and the mean eGFR was 25 + 11 mL/min/1.73m^2^. Frailty was diagnosed in 51 patients, and 40 patients were classified as pre-frail. AGEs and RAGE isoforms seem not to correlate with overall frailty. Instead, AGEs were associated with specific frailty domains, inversely associated with BMI (R = −0.22, *p* = 0.016) and directly associated with gait test time (R = 0.17, *p* = 0.049). AGEs were also associated with involuntary weight loss (OR 1.84 *p* = 0.027), independent of age and sex. Conclusions: AGEs are associated with some pivotal components of the frailty phenotype, although they are not associated with frailty overall.

## 1. Introduction

Frailty is defined as a decline in functional reserve and resistance to stressors across multiorgan systems that arises during the aging process [1,2,3]. Chronic kidney disease is known as one of the most representative conditions that can accelerate premature aging [2,4]. Chronic inflammation, insulin resistance and increased uremic toxins, all conditions associated with CKD, have been shown to contribute to the risk of frailty [2,5,6,7]. In elderly patients with CKD, frailty is highly prevalent compared to those with normal renal function [8,9], and its prevalence is inversely proportional to renal function [9,10]. Furthermore, frail patients are 2.5 times as likely to die or initiate dialysis than non-frail patients after accounting for initial kidney function and comorbidity [11].

Advanced glycation end products (AGEs) derive from the Maillard reaction with non-enzymatic modifications of the amino groups and polyol pathways of proteins or lipids by reducing sugars and their metabolites [12,13]. Several environmental factors, such as smoke; bad dietary habits, including consumption of high doses of carbohydrates doses and highly processed foods; a hypercaloric diet; and a sedentary lifestyle, can induce AGE production [14]. In CKD, the accumulation of AGEs is the result of to two mechanisms: reduced renal clearance and increased production, which is the net result of an imbalance between oxidant/antioxidant homeostasis [15,16,17].

One of the main causes of frailty in CKD patients is malnutrition [12,18]. The etiology of malnutrition in chronic kidney disease is multifactorial: reduction in food intake consequent to anorexia, changes in taste, uremic gastritis and a high number of prescribed medications may all contribute to reducing protein and energy intake [18,19,20]. Moreover, in end-stage renal disease, AGE levels have been associated with the incidence of malnutrition [2]. Indeed, AGEs are implicated in several pathophysiologic mechanisms that can promote involuntary weight loss, such as increased inflammation, enhanced protein catabolism and energy expenditure [2,21].

Sarcopenia is often a precursor of frailty [22]. AGEs have been hypothesized to play a role also in the pathogenesis of sarcopenia through AGE-mediated increases in inflammation and endothelial dysfunction in the skeletal muscle microcirculation [22,23,24], as well as through crosslinking of collagen in skeletal muscle [23,25]. High AGE levels are also associated with poor handgrip strength and slow walking speed, two dysfunctions that are strongly correlated with frailty [23,26]. Furthermore, high AGE serum levels are associated with prevalent frailty in older adults [27]. An in vitro study showed that AGEs can induce muscle atrophy and impair myogenesis through RAGE-mediated signaling [28].

Given these premises, we hypothesize that the accumulation of AGEs might contribute to the pathogenesis of frailty in patients with advanced CKD. Therefore, in this study, we explored whether serum AGEs and the different RAGE isoforms of soluble RAGE (sRAGE), i.e., cleaved RAGE (cRAGE) and endogenous secretory RAGE (esRAGE), are associated with frailty in this population. 

## 2. Materials and Methods

### 2.1. Patients and Study Design

In this cross-sectional study, we enrolled 117 prevalent patients between September 2016 and March 2018. We applied the following selection criteria: age ≥65 years, CKD stages 3a to 5, in conservative therapy, and relatively stable eGFR over the previous 6 months (with less than 2 mL/min/1.73/m^2^ of variation). eGFR was estimated according to the CKD-EPI formula. To eliminate possible confounding factors, we excluded patients with cancer, cirrhosis and/or ascites, severe heart failure (NYHA class III–IV), nephrotic syndrome, thyroid diseases, bowel inflammatory diseases and inability to cooperate. We also excluded patients taking immunosuppressive drugs and those who had been hospitalized in the last three months. Urinary and biochemical parameters were collected during a morning visit after an overnight fasting of at least 12 h. All patients signed an informed consent form, and the study was conducted according to the ICP Good Clinical Practices Guidelines. The study was approved by the Ethics Committee of our institution (Milano 2; approval 347/2010). 

### 2.2. sRAGE, esRAGE and cRAGE Quantification

sRAGE and its isoforms were measured as previously described [29]. In short, sRAGE and esRAGE were quantified using two ELISA kits: sRAGE with a kit from R&D Systems (DY1145, Minneapolis, MN, USA) and esRAGE with a kit from B-Bridged International (K1009–1, Santa Clara, CA, USA). The intra- and interassay coefficients of variation of the esRAGE assay were 6.37 and 4.78–8.97%, respectively. cRAGE levels were obtained by subtracting esRAGE from total sRAGE; then, we calculated the AGE/sRAGE ratio. We used a GloMax^®^ multi microplate multimode reader (Promega, Milan, Italy) to perform photometric measurements. 

### 2.3. AGE Quantification

We quantified AGE levels using a fluorescence spectrophotometer (GloMax^®^, Promega, Milan, Italy). The fluorescence intensity of plasma samples was measured at 414–445 nm after excitation at 365 nm, as previously reported [30,31]. Fluorescence intensity was expressed in arbitrary units (A.U.). AGEs were then normalized for total protein content. The average inter- and intraassay CV of fluorescent AGEs were 7.3% and 5.99%, respectively.

### 2.4. Frailty Assessment

To assess frailty, we used the frailty phenotype (FP) proposed by Fried and colleagues [32]. In brief, the following five components were used to assess frailty: (1) involuntary weight loss ≥4.5 kg in 12 months; (2) exhaustion, defined as feeling tired ≥4 days per week for more than 3 months; (3) weakness, defined as a handgrip strength <16 kg in females and <27 kg in males; (4) slowness, defined as a 4 m course gait test speed >0.8 m/s; reduced physical activity, defined as a score <7 on the physical activity scale [1]. Patients with three or more deranged items were classified as frail.

### 2.5. Anthropometric Measurements

We determined body weight, height, and body mass index (BMI), according to the Quetelet index (kg/m^2^). 

### 2.6. Statistical Analysis

We expressed continuous variables as mean with standard deviation (SD) for parametric data or as median with interquartile range (IQR) for non-parametric distribution. Categorical variables were expressed as percentages. We performed Student’s t-test and ANOVA to compare parametric variables, and when appropriate, we used the Mann–Whitney “U” test or Kruskal–Wallis for non-parametric variables.

A general linear model (GLM) was used to test the correlation between frailty domains, AGEs and RAGE isoforms. 

Statistical analysis was conducted using IBM SPSS software (version 25, IBM, Armonk, NY, USA).

## 3. Results

### 3.1. General Population Characteristics

In our study, we enrolled 117 patients, the general characteristics of whom are depicted in Table 1. The mean age of the patients was 80 ±11 years. Most patients were male. Nearly half of patients had diabetes (56%), and, on average, they were overweight, with a mean BMI of 28 ± 5 kg/m^2^. Most of the patients in our cohort were classified as frail (44%), almost a third could be labeled as intermediate or pre-frail (33%) and only a minority could be defined as non-frail (22%). eGFR ranged from 60 to 8 mL/min/1.73 m^2^, with a mean value of 25 ± 11 mL/min/1.73 m^2^ (stage 4b CKD). 

Average nutritional metabolic markers were normal (total cholesterol, 168 ± 37 mg/dL; albumin, 4.0 ± 0.4 g/dL; prealbumin, 28 ± 5 mg/dL).

### 3.2. General Cohort Characteristics, Inflammation and Frailty

As evident in Table 2, frail patients were significantly older, especially when compared to non-frail patients (81 ± 6 vs. 66 ± 18, *p* < 0.0001). In general, male patients were preponderant in the non-frail and pre-frail groups compared to frail individuals (88% for pre-frail vs. 88% for non-frail and 47% for established frailty; *p* < 0.0001), and frail patients had lower creatinine clearance than non-frail and pre-frail patients (20.8 (16.6–28.0) vs. 34.3 (20.8–45.1) and 30.6 (16.6–36.6), respectively; *p* = 0.002). No significant differences were noted according to frailty status in regard to diabetes and BMI.

Concerning metabolic parameters, the main alterations were noticeable in regard to factors related to patient nutritional status. Prealbumin, albumin and hemoglobin levels were significantly lower in frail patients, especially when compared to non-frail patients (prealbumin, 29.2 ± 5.6 vs. 26.8 ± 5, *p* = 0.011; albumin, 4.3 ± 0.4 vs. 4 ± 0.3, *p* = 0.002; hemoglobin, 13.1 ± 1.8 vs. 12 ± 1.3, *p* = 0.003). 

Among inflammatory markers, only TNFα showed significantly increased levels in frail patients compared to non-frail patients (non-frail, 11.3 ± 8.8 vs. frail, 16.1 ± 8.3; *p* = 0.04) 

### 3.3. Variation in AGEs and Different RAGE Isoforms According to Frailty Status

As depicted in Table 3, no significant variation in AGE levels was observed in relation to different frailty status in our cohort. Even when the AGs/sRAGE ratio was taken into account, no significant variations were observed. The same was true when considering RAGE isoforms. 

We also performed an analysis assessing the eventual relationship between the cRAGE/esRAGE ratio and frailty status; however, in this case, no evident variation was found.

### 3.4. Variation in AGEs and RAGE Isoforms According to Different Frailty Domains

We deepened our analysis by evaluating the eventual correlation between AGEs and different RAGE isoforms with the single domains that define the phenotype of frailty.

As evident in Table S1 in the Supplementary Materials**,** AGEs were only correlated with involuntary weight loss. Moreover, a correlation of borderline significance was found between cRAGE and patient exhaustion.

### 3.5. Evaluation of the Association between AGEs and RAGE Isoforms and Frailty Domains

**Figure 1:**
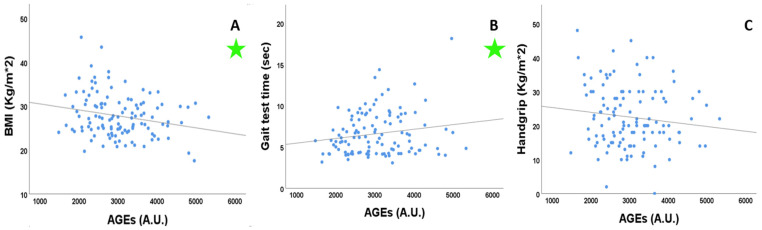
Linear regression analyses of the association of AGEs with BMI, gait speed and hand grip. (**A**) R = −0.22, *p* = 0.016; (**B**) R = 0.17, *p* = 0.049; (**C**) R = −0.124, *p* = 0.18. BMI: body mass index; AGEs: advanced glycation end products; A.U.: arbitrary unit. Significant regressions are marked with a green star.

We performed a linear regression analysis to evaluate the eventual association between AGEs and different RAGE isoforms with BMI handgrip strength and gait test time (Figure 1). AGE levels showed an inverse and significant association with BMI (R= −0.22, *p* = 0.016) and a direct and significant association with gait test time (R = 0.17, *p* = 0.049). 

We then employed various multivariate linear regression models in which we weighted the correlations of AGEs and RAGEs with frailty domains for creatinine clearance and patient age.

The significant results of these analyses are shown in detail in Tables S3 and S4. When weighted for creatinine clearance, AGE/RAGE levels were independently and inversely associated with lower BMI (B = −0.176, *p* = 0.049) (Table S3). When the model was corrected for patient age instead of creatinine clearance (Table S4), AGEs were still inversely and independently associated with BMI (B = −0.183, *p* = 0.049). AGEs were also directly associated with gait test time (B = 0.142, *p* = 0.06), although with a borderline statistical significance. The AGE/sRAGE ratio was associated with patient BMI (inverse proportionality, B = −0.156, *p* = 0.06), even if the relationship was of borderline significance. 

To confirm the strength of the association between AGEs and weight loss (frailty domain), we employed two multivariate analysis models. In the first model, we considered weight loss as a categorical frailty domain and included age, sex and creatinine clearance, which we found to be the main confounders in this context. As evident in Table 4, the relationship between AGEs and weight loss remained significant when age, sex or both were included in the model (AGEs_(sex, age)_ OR 1.84 (1.1–3.5), *p* = 0.027; AGEs_(sex)_ 1.84 (1.3–4.1), *p* = 0.019; AGEs_(age)_ OR 1.91 (1.06–3.5), *p* = 0.0028). Instead, the association of AGEs with weight loss was non-significant when creatinine clearance was included in the model. We also performed a multivariate linear regression considering BMI as a continuous equivalent of the weight loss domain, as shown in Table 5. We corrected AGEs for sex, age and creatinine clearance. We found that the significant inverse association between AGEs and BMI was maintained when age, sex or both were included in the model, whereas the relationship was of borderline significance when creatinine clearance was added to the model (AGEs_(sex. age)_ B −0.23, *p* = 0.01; AGEs_(cr.cl. age)_ B −0.17, *p* = 0.049; AGEs_(age)_ B −0.23, *p* = 0.01; AGEs_(cr.cl.)_ B −0.18, *p* = 0.048; AGEs_(age)_ B −0.23, *p* = 0.01). The association between AGEs and BMI was lost when all the variables were considered together (AGEs_(cr.cl, sex, age)_ B 0.13, *p* = 0.17). 

## 4. Discussion

The main findings of our paper can be summarized as follows: AGEs were not correlated with overall frailty but were associated with specific frailty domains. In particular, unintentional weight loss was the frailty domain mainly influenced by AGE levels, and AGEs were the main factor associated with lower BMI, even after correction for other potential confounders, such as creatinine clearance. The association of AGEs with involuntary weight loss was lost when creatinine clearance was included in the model, whereas this association was maintained independent of age or sex. This may indicate that AGEs are increased in e individuals with lower creatine clearance and that this increase is independent of age and sex. Moreover, in our multivariate analysis, the correlation of AGEs with BMI, which was independent of age and creatinine clearance, taken alone was weakened to borderline significance when both of these variables were included in the model. Our data suggest that, in older subjects affected by advanced CKD, AGEs are associated with a poor nutritional status and that their accumulation (possibly because of a decrease in renal function) may influence unintentional weight loss independent of age. These results support the independent association of AGEs with poor nutritional status that was previously described by our group in older patients with advanced CKD [33]. The cross-sectional design of our study prevents us from demonstrating any causal correlation between AGEs, unintentional weight loss and BMI.

The hypothesis of the systemic effect of AGEs is supported by previously published evidence that seems to link AGEs to the development of several components of the frailty phenotype.

The most likely explanation for the detrimental effect of AGEs on patients’ global functioning could be that AGE accumulation in tissue could reflect the overall toxin and oxidative-stress burden of body structures, serving as a sort of “metabolic memory” of systemic aging [34]. 

Regarding the impact of AGEs on muscles, higher AGE serum levels and tissue deposition seem to be associated with the development of sarcopenia, which has a major role in the establishment of the frailty phenotype; this hypothesis also seems to be valid for CKD patients [13,35,36]. AGEs can have negative effects on several aspects of muscle biomechanical functions, inducing atrophy [28], increased muscle stiffness [25] and impaired muscle fiber function [20,24,37,38]. Finally, in a recently published study performed by our group, AGE levels were associated with sarcopenia independent of eGFR and were linearly associated with gait test time, a marker of muscular function [12]. 

Another important component of the frailty phenotype that appears to be influenced by AGEs is patient nutritional status. In a recent study by Suliman et al. conducted on patients with ESRD, higher levels of an AGE subtype, pentosidine, were associated with inflammation and the onset of malnutrition [39,40,41]. Probably the most important result on this matter was obtained by Viramontes et.al, who observed that patients undergoing dialysis who died during the follow-up showed significantly higher AGE levels and were significantly more malnourished than other patients [42]. Finally, in a recent study conducted in our center, RAGE levels were associated with malnutrition independent of eGFR [33].

The link between AGEs and malnutrition development could be explained by the possible association between AGE levels and the rise in proinflammatory cytokines, which may induce anorexia and protein-catabolic status [43,44,45,46].

Our study is one of the few works currently available in literature addressing the relationship between AGEs and frailty, and our results are in accordance with most previous studies. First of all, in a review performed by Semba et al. in 2010, a literature analysis supported the hypothesis that AGEs may be an important factor in the progression of the overall aging process [47].

In a study by Whitson et.al performed on 3373 patients, a particular AGE, CML (carboxymethyl-lysine), showed a correlation with frailty development [32]. In this study, the association between CML and the development of frailty remained significative until eGFR was added to the model. In particular, CML was associated mainly with three frailty domains: reduced strength, exhaustion and low physical activity [23]. Analogous data were provided by Semba et. al. from the CHIANTI study cohort. In this case, CML was directly and independently associated with slow walking speed [27]. These results overlap with those derived from another study focusing on elderly women, in which higher CML levels were independently associated with impaired walking speed [48]. Another study from the same cohort confirmed our observation of the possible impact of AGEs on patient BMI. Higher CML levels were inversely associated with patient total fat mass, even after adjustment for renal function [21].

Other important results were reported in a recent prospective study performed by Pilleron et al. [49]. In this study, incident frailty was independently associated with AGEs, as evaluated by skin autofluorescence, even after adjustment for diabetes and CKD [50]. 

Finally, a recent study by Mahmoudy et al. showed that skin autofluorescence and homocitrulline were independently associated with an increased risk of frailty development [46], even after correction for eGFR; however, other forms of AGEs did not show the same association [51]. The divergence relative to the results obtained in our study could be due to the fact that in this case, single forms of AGEs were taken into account, which may show specific relationship that may be lost in a pooled analysis [52].

We acknowledge that our study has some limitations. First of all, the cross-sectional design does not allow for attribution of any causal relationship to the association between AGEs levels and the onset of frailty in CKD patients. Secondly, our study is monocentric, and our population is relatively small. However, the monocentric nature of our study allowed us to reduce the possible sources of bias by using a highly standardized protocol for patient selection, biochemical analyses and clinical observations. In particular, we applied strict inclusion and exclusion criteria that let us exclude patients who may have developed frailty because of specific clinical conditions. Lastly, quantification of fluorescent AGEs and the lack of analyses addressing the role of particular AGE isoforms could have masked the associations between specific AGEs and frailty or its components. More than 20 different AGEs have been identified in human blood and tissues. According to their chemical properties, they can be classified as fluorescent or non-fluorescent AGEs. Due to their great heterogeneity, there is not a single test for their overall quantification. Although mass spectrometric analysis is highly promising for AGE detection, its use is not widespread. In recent years, novel in vivo and non-invasive spectroscopic methods that measure the autofluorescence of AGEs have been developed, and the quantification of skin autofluorescence as a marker of AGE accumulation has been introduced as a non-invasive method in clinical practice [53]. The same detection method can be used on plasma. Although this method cannot identify the specific contribution of individual AGEs as pathogenetic molecules and/or biomarkers, it allows for the cumulative quantification of multiple compounds, providing important information about the total amount of AGEs that are produced. Therefore, the overall impact of AGE accumulation on the development of frailty in older CKD patients can be summarized. A significant point of strength of our study is that the relationship between AGEs, RAGEs and the frailty phenotype was thoroughly investigated. Furthermore, we studied the association between AGEs and frailty in depth by addressing not only AGEs but also RAGE isoforms and the modulation of the interaction between AGEs and RAGEs in a proinflammatory milieu.

This is, to the best of our knowledge, the first time that a comprehensive evaluation of the association between the AGEs–RAGE system and frailty has been conducted in older CKD patients not undergoing dialysis. 

## 5. Conclusions

AGEs are not associated with the globality of the frailty phenotype, but they are independently associated with involuntary weight loss, which is a main component of frailty. 

Further studies are needed to clarify these links, focusing on markers of tissue AGE deposition (SAF), specific AGE isoforms and different, more comprehensive frailty definitions. Moreover, prospective analyses are needed to strengthen these observations. 

## Figures and Tables

**Table 1:** General cohort characteristics.

Variable	Overall Cohort (n = 117)
*General characteristics*	
Age (years)	80 ± 11
Males/females, n (%)	82 (70)/35 (30)
Diabetes, n (%)	65 (56)
BMI, (kg/m^2^)	28 ± 5
Frailty	
Not frail, n (%)	26 (22)
Pre-frail; n (%)	40 (34)
Frail, n (%)	51 (44)
*Metabolic characteristics*	
eGFR, (mL/min/1.73m^2^)	25 ± 11
Creatinine clearance (mL/min)	24.4 [17.2–36.0]
Uric acid (mg/dL)	6.0 ± 1.5
Total cholesterol (mg/dL)	168 ± 37
HDL cholesterol (mg/dL)	51 ± 15
Triglycerides (mg/dL)	130 ± 54
Albumin (g/dL)	4.0 ± 0.4
Prealbumin (mg/dL)	28 ± 5
Proteinuria 24 h (g/24 h)	1.2 ± 1.6
*Inflammatory status*	
CRP (mg/dL)	0.4 ± 0.7
TNF alpha (pg/mL)	15.3 ± 8.2

eGFR, estimated glomerular filtration rate; BMI, body mass index; LDL: low-density lipoprotein; HDL: high-density lipoprotein; CRP, c-reactive protein. Data are expressed as mean with standard deviation or as number and percentages.

**Table 2:** Comparison of general characteristics and metabolic parameters between non-frail, pre-frail and frail patients.

Variables	Non-Frail (n = 26)	Pre-Frail (n = 40)	Frail (n = 51)	*p*
Males, n (%)	23 (88)	35 (88)	24 (47)	**<0.0001**
Diabetes, n (%)	13 (50)	23 (58)	29 (57)	0.8
Age, (years)	66 ± 18	79 ± 6	81 ± 6	**<0.0001**
BMI, (kg/m^2^)	26.1 ± 3.6	27.9 ± 4.5	28.5 ± 5.6	0.13
*Metabolic characteristics*				
eGFR, (mL/min/1.73 m^2^)	28.8 ± 13.6	23.4 ± 10.3	23.8 ± 9.7	0.097
Creatinine clearance, (mL/min)	34.3 [20.8–45.1]	30.6 [16.6–36.6]	20.8 [16.6–28.0]	**0.002**
Total cholesterol, (mg/dL)	165.4 ± 30.2	167.0 ± 33.8	170.9 ± 42.8	0.79
HDL cholesterol, (mg/dL)	53.7 ± 19.5	50.9 ± 13.0	54.8 ± 21.1	0.59
Triglycerides, (mg/dL)	111.8 ± 40.9	135.8 ± 57.2	132.0 ± 55.9	0.18
Uric acid, (mg/dL)	6.3 ± 1.3	6.0 ± 1.4	6.1 ± 1.7	0.83
Prealbumin, (mg/dL)	29.2 ± 5.6	30.1 ± 5.4	26.8 ± 5.0	**0.011**
Albumin, (g/dL)	4.3 ± 0.4	4.1 ± 0.3	4.0 ± 0.3	**0.002**
Hb, (g/dL)	13.1 ± 1.8	12.8 ± 1.4	12.0 ± 1.3	**0.003**
Urinary protein (mg/24 h)	882 ± 899	1088 ± 1337	1314 ± 1886	0.5
*Inflammatory markers*				
CRP, (mg/dL)	0.4 ± 0.9	0.5 ± 0.7	0.5 ± 0.7	0.96
TNFα, (pg/mL)	11.3 ± 8.8	13.8 ± 5.4	16.1 ± 8.3	**0.04**

BMI: body mass index; eGFR: estimated glomerular filtration rate; HDL: high-density lipoprotein; Hb: hemoglobin; CRP: c-reactive protein; TNFα: tumor necrosis factor alpha. Data are expressed as mean with standard deviation. *p* values identify trends, and values less than 0.05 are indicated in bold.

**Table 3:** Concentration of AGEs and sRAGE isoforms in non-frail, pre-frail and frail CKD patients.

Variable	Not Frail (n = 26)	Pre-Frail (n = 40)	Frail (n = 51)	*p*
AGEs, (arbitrary units)	2932 ± 912	2997 ± 822	3086 ± 732	0.71
sRAGE, (pg/mL)	2291 ± 1131	2144 ± 1205	2551 ± 1380	0.31
esRAGE, (pg/mL)	541 [369–737]	591 [403–814]	468 [360–668]	0.63
cRAGE, (pg/mL)	1649 ± 805	1521 ± 777	1856.8 ± 1071	0.22
AGEs/sRAGE, (arbitrary units)	1.7 ± 1.2	1.8 ± 0.9	1.5 ± 0.8	0.52
cRAGE/esRAGE, (arbitrary units)	2.8 ± 0.9	2.8 ± 1.1	2.7 ± 0.7	0.78

AGEs: advanced glycation end products; sRAGE: soluble receptor for AGE; esRAGE: endogenous secretory receptor for AGE; cRAGE: cleaved receptor for AGE; CKD: chronic kidney disease. Data are expressed as mean with standard deviation. *p* values less than 0.05 are indicated in bold.

**Table 4:** Multivariate logistic regression analysis of the association between AGEs and involuntary weight loss weighted for sex, age and creatinine clearance.

Dependent Variable	Variable	OR	*p*
*Involuntary weight loss*	Sex	1.83	0.30
	AGEs (A.U.)	1.65	0.108
	Cr. Cl (mL/min)	0.98	0.35
	Age (years)	**1.08**	**0.049**
	Sex	1.70	0.36
	AGEs (A.U.)	**1.84**	**0.027**
	Age (years)	**1.08**	**0.035**
	Sex	1.76	0.33
	AGEs (A.U.)	1.59	0.14
	Cr. Cl (mL/min)	0.97	0.20
	AGEs (A.U)	1.61	0.10
	Cr. Cl (mL/min)	0.98	0.42
	Age (years)	**1.07**	**0.05**
	Sex	1.55	0.44
	AGEs (A.U.)	**1.84**	**0.019**
	AGEs (A.U.)	1.69	0.13
	Cr. Cl. (mL/min)	0.97	0.25
	AGEs (A.U.)	**1.911**	**0.0028**
	Age (years)	**1.08**	**0.039**

AGEs, advanced glycation end products; Cr. Cl., creatinine clearance; A.U., arbitrary unit; OR, odds ratio. *p* values less than 0.05 and corresponding OR are indicated in bold.

**Table 5:** Multivariate linear regression analyses testing the association of AGEs with BMI.

Dependent Variable	Variable	B	*p*
*BMI (kg/m^2^)*	Overall	0.27	0.06
	Sex	0.06	0.47
	AGEs (A.U.)	0.13	0.17
	Cr. Cl (mL/min)	0.13	0.21
	Age (years)	0.13	0.17
	Overall	**0.25**	**0.05**
	Sex	0.05	0.59
	AGEs (A.U.)	**−0.23**	**0.01**
	Age (years)	0.10	0.25
	Overall	0.25	0.07
	Sex	0.07	0.43
	AGEs (A.U.)	**−0.17**	**0.05**
	Cr. Cl (mL/min)	0.10	0.32
	Overall	0.28	0.02
	AGEs (A.U)	**−0.17**	**0.049**
	Cr. Cl (mL/min)	0.12	0.24
	Age (years)	0.15	0.08
	Overall	0.23	0.04
	Sex	0.06	0.53
	AGEs (A.U.)	**−0.22**	**0.01**
	Overall	**0.23**	**0.03**
	AGEs (A.U.)	**−0.18**	**0.048**
	Cr. Cl. (mL/min)	0.09	0.38
	Overall	**0.26**	**0.01**
	AGEs (A.U.)	**−0.23**	**0.01**
	Age (years)	0.14	0.12

AGEs, advanced glycation end products; Cr. Cl., creatinine clearance; A.U., arbitrary unit. *p* values less than 0.05 and corresponding B values are indicated in bold.

## Data Availability

The dataset analyzed for this study can be found in the OSF repository at https://osf.io/qtch5/?view_only=bf0a2aa093854cc689d7c7eee979dfa9, accessed on 15 October 2022.

## Supplementary Materials

The following supporting information can be downloaded at: https://www.mdpi.com/article/10.3390/cells12030438/s1, Table S1: General and metabolic variables classified according to the presence (yes) or absence (not) of alterations in the frailty domains; Table S2: Concentration of inflammatory markers and uremic toxins classified according to the presence (yes) or absence (no) of alterations in the frailty domains; Table S3: Linear regression analyses of the association of AGEs and RAGEs with BMI, gait speed and hand grip weighted for eGFR; Table S4: Linear regression analyses of the association of AGEs and RAGEs with BMI, gait speed and hand grip weighted for age.
