# Supplementary material for: Association of Autofluorescent Advanced Glycation End Products (AGEs) with Frailty Components in Chronic Kidney Disease (CKD): Data from a Single-Center Cohort Study
Source: Cells. 2023 Jan 29;12(3):438. doi: 10.3390/cells12030438 (PMC9913604; doi:10.3390/cells12030438)
Supplement: Supplementary file 1 [file cells-12-00438-s001.zip › cells-2136147-SI.pdf]

Table S1. Concentration of AGEs and sRAGE isoforms classified according to the presence (yes) or absence (not) of alterations in the frailty domains.

Frailty Domain	Variables	Not	Yes	*p*
Weight Loss	AGEs (arbitrary unit)	2939±781	3400±805	**0.016**
	sRAGE (pg/mL)	2331±1234	2463±1463	0.67
	esRAGE (pg/mL)	536 [382-718]	589 [361-828]	0.29
	cRAGE (pg/mL)	1695±895	1693±1079	0.99
	AGEs/sRAGE (arbitrary unit)	1.6±0.9	1.9±1.2	0.23
	cRAGE/esRAGE (arbitrary unit)	2.8±0.9	2.6±1.1	0.27
Weakness	AGEs (arbitrary unit)	2937±836	3073±781	0.37
	sRAGE (pg/mL)	2147±1198	2480±1307	0.17
	esRAGE (pg/mL)	467 [336-719]	565 [418-761]	0.16
	cRAGE (pg/mL)	1561±865	1776±957	0.22
	AGEs/sRAGE (arbitrary unit)	2±1	2±1	0.13
	cRAGE/esRAGE (arbitrary unit)	2.8±0.8	2.8±1	0.94
Exhaustion	AGEs (arbitrary unit)	2995±863	3057±721	0.68
	sRAGE (pg/mL)	2182±1163	2579±1381	0.09
	esRAGE (pg/mL)	486 [361-717]	600 [402-895]	0.31
	cRAGE (pg/mL)	1554±814	1880±1034	0.06
	AGEs/sRAGE (arbitrary unit)	2±1	2±1	0.26
	cRAGE/esRAGE (arbitrary unit)	2.6±0.8	2.7±1	0.75
Slowness	AGEs (arbitrary unit)	2978±860	3091±702	0.46
	sRAGE (pg/mL)	2336±1293	2383±1252	0.85
	esRAGE (pg/mL)	520 [365-747]	555 [407-715]	0.59
	cRAGE (pg/mL)	1648±879	1770±1002	0.50
	AGEs/sRAGE (arbitrary unit)	1.7±1.0	1.6±0.8	0.69
	cRAGE/esRAGE (arbitrary unit)	2.7±0.9	2.9±1	0.39
Low physical activity level	AGEs (arbitrary unit)	3018±821	3027±786	0.95
	sRAGE (pg/mL)	2273±1143	2449±1413	0.46
	esRAGE (pg/mL)	493 [389-716]	561 [353-803]	0.74
	cRAGE (pg/mL)	1619±764	1784±1088	0.34
	AGEs/sRAGE (arbitrary unit)	1.7±1.0	1.6±0.9	0.76
	cRAGE/esRAGE (arbitrary unit)	2.7±0.8	2.9±1.1	0.31

AGEs: Advanced Glycation End products; sRAGE: soluble receptor for AGE; esRAGE: endogenous secretory receptor for AGE; cRAGE: cleaved receptor for AGE. Data are expressed as mean with standard deviation. p values less than 0.05 are indicated in bold.

Table S2. Concentration of inflammatory markers and uremic toxins classified according to the presence (yes) or absence (not) of alterations in the frailty domains.

Frailty Domain	Variables	Not	Yes	*p*
Weight Loss	CRP, (mg/dL)	0.4±0.7	0.5±1.0	0.51
	TNFα, (pg/mL)	14.0±7.8	15.3±7.5	0.53
Weakness	CRP, (mg/dL)	0.4±0.7	0.5±0.8	0.75
	TNFα, (pg/mL)	11.7±7.6	15.8±7.4	**0.006**
Exhaustion	CRP, (mg/dL)	0.5±0.9	0.4±0.5	0.47
	TNFα, (pg/mL)	13.8±8.4	14.7±6.8	0.55
Slowness	CRP, (mg/dL)	0.4±0.7	0.5±0.8	0.78
	TNFα, (pg/mL)	12.9±7.1	16.2±8.3	**0.033**
Low physical activity level	CRP, (mg/dL)	0.4±0.6	0.6±0.8	0.16
	TNFα, (pg/mL)	13.5±7.6	15±7.9	0.32

CRP: c-reactive protein; TNFα: Tumor necrosis factor alpha. Data are expressed as mean with standard. *p* values are intended for trend and values less than 0.05 are indicated in bold.

Table S3. Linear regression analyses of the association of AGEs and RAGEs with BMI, gait speed and handgrip weighted for creatinine clearance.

Dependent variable	Variables	B	*p*
	**R= 0.215 p=0.047**		
	AGEs, (A.U.)	-0.127	0.19
	Cr. Cl. (mL/min)	0.119	0.22
	**R= 0.24 p=0.03**		
	AGEs/sRAGE	**-0.176**	**0.049**
BMI (kg/m²)	Cr. Cl. (mL/min)	**0.196**	**0.03**
	**R= 0.213 p=0.048**		
	sRAGE (A.U.)	0.11	0.26
	Cr. Cl. (mL/min)	**0.228**	**0.02**
	**R= 0.174 p=0.18**		
	AGEs, (A.U.)	**0.159**	**0.06**
	Cr. Cl. (mL/min)	-0.029	0.72
	**R= 0.131 p=0.38**		
	AGEs/sRAGE	0.067	0.48
Gait test time (s)	Cr. Cl. (mL/min)	-0.117	0.216
	**R= 0.130 p=0.37**		
	sRAGE (A.U.)	0.051	0.57
	Cr. Cl. (mL/min)	**0.318**	**0.001**
	**R= 0.198 p=0.10**		
	AGEs, (A.U.)	-0.026	0.8
	Cr. Cl. (mL/min)	0.183	0.09
	**R= 0.225 p=0.045**		
	AGEs/sRAGE	-0.1	0.14
Handgrip strenght (kg/m²)	Cr. Cl. (mL/min)	**0.203**	**0.02**
	**R= 0.201 p=0.07**		
	sRAGE (A.U.)	0.077	0.44
	Cr. Cl. (mL/min)	**0.225**	**0.04**

BMI: Body Mass Index; AGEs: Advanced Glycation End products; sRAGE: soluble receptor for AGE; Cr. Cl., creatinine clearance; A.U.: Arbitrary unit.

Table S4. Linear regression analyses of the association of AGEs and RAGEs with BMI, gait test and handgrip strength weighted for age.

Dependent variable	Variables	B	*p*
	**R= 0.223 p=0.042**		
	AGEs, (A.U.)	**-0.183**	**0.049**
	Age, (years)	-0.072	0.43
	**R= 0.161 p=0.22**		
	AGEs/sRAGE	**-0.156**	**0.06**
BMI (kg/m²)	Age, (years)	-0.073	0.43
	**R= 0.093 p=0.613**		
	sRAGE (A.U.)	0.023	0.80
	Age, (years)	-0.089	0.34
	R=**0.347 p=0.001**		
	AGEs, (A.U.)	**0.142**	**0.06**
Gait test time (s)	Age, (years)	**0.303**	**0.001**

	R=**0.318 p=0.003**		
	AGEs/sRAGE	0.02	0.82
	Age, (years)	**0.314**	**0.001**
	R=**0.321 p=0.002**		
	sRAGE (A.U.)	0.051	0.57
	Age, (years)	**0.318**	**0.001**
Handgrip strenght (kg/m²)	R=**0.566 p=0.000**		
	AGEs, (A.U.)	-0.071	0.36
	Age, (years)	**-0.554**	**0.000**
	R=**0.561 p=0.000**		
	AGEs/sRAGE	-0.02	0.8
	Age, (years)	**-0.559**	**0.000**
	R=**0.562 p=0.000**		
	sRAGE (A.U.)	-0.026	0.74
	Age, (years)	**-0.562**	**0.000**

AGEs: Advanced Glycation End products; sRAGE: soluble receptor for AGE; A.U.: Arbitrary unit.
